# Preliminary Field Performance of a Low-Tortuosity Permeable Pavement System Incorporating Bottom Ash Fine Aggregate for Surface-Temperature Regulation and Stormwater Storage

**DOI:** 10.3390/ma19153189

**Published:** 2026-07-26

**Authors:** Chan-Gi Park, Ri-On Oh, Sang-Hyeon Park, Sung-Ki Park, Hwang-Hee Kim, Derick Gabriel Stein, Jaeheum Yeon

**Affiliations:** 1 Department of Regional Construction Engineering, Kongju National University, Yesan 32439, Republic of Korea; cgpark@kongju.ac.kr; 2 Research Center, Contecheng Co., Ltd., Namyangju 12248, Republic of Korea; ohrion@contecheng.co.kr (R.-O.O.); wnahd4989@gmail.com (S.-H.P.); skpark@contecheng.co.kr (S.-K.P.); 3 Research Center, GKtech Co., Ltd., Namyangju 12245, Republic of Korea; hwang1032@gktech.co.kr; 4 Interdisciplinary Program in Earth Environmental System Science & Engineering, Kangwon National University, Chuncheon 24341, Republic of Korea; gabriel.stein@kangwon.ac.kr; 5 Department of Regional Infrastructure Engineering, Kangwon National University, Chuncheon 24341, Republic of Korea

**Keywords:** low-tortuosity permeable pavement, bottom ash, urban heat island effect, stormwater runoff, low-impact development

## Abstract

**Highlights:**

- A pilot field evaluation was conducted on a prototype permeable pavement block system.
- The prototype interlocks bottom ash pavers with low-tortuosity flow paths and subsurface storage blocks.
- ROI-based thermal imaging indicated modest surface-temperature regulation.
- Simulated rainfall provided qualitative evidence of on-site water entry and temporary storage.
- Theoretical storage assessment indicates the potential of the pavement system to further delay runoff.

**Abstract:**

Rapid urbanization has intensified two critical urban challenges: the urban heat island effect and stormwater runoff. This study evaluates the pilot-level field performance of a low-tortuosity permeable pavement (LTPP) system in potentially contributing to improved thermal regulation and hydraulic functionality. The system comprises a reduced-tortuosity upper block incorporated with bottom ash (BA) as a recycled fine aggregate and an underlying storage unit connected through an interlocking configuration, enabling direct infiltration while reducing clogging susceptibility and improving resistance to settlement and displacement. Field tests included thermal imaging, water-spraying infiltration-storage and vehicle-loading observations, and theoretical storage analysis. Initially, conventional permeable pavement (PP) dry surface temperature was measured at 44.2 °C, whereas the LTPP system already exhibited a lower temperature of 42.4 °C. During the evaporative stage after wetting, the LTPP system showed a lower temperature recovery rate, with a 2.91% increase between 90 and 120 min compared with 3.60% for conventional permeable pavement, indicating improved surface-temperature regulation. The storage calculations approximated that the LTPP system could theoretically buffer the simulated 15.63 mm/h rainfall by 6.65 to 7.32 h. It was also determined using historical rainfall data that the LTPP system, especially when provided with an outlet or drainage system, could effectively accommodate short- to medium-duration rainfall. Water-spraying tests confirmed rapid infiltration and subsurface storage, while vehicle-loading observations showed no noticeable displacement or settlement. These findings highlight the potential of a multifunctional permeable pavement design strategy that combines low-tortuosity flow paths, functional recycled aggregate selection, and subsurface storage for surface-temperature regulation and stormwater management.

## 1. Introduction

The urban heat island (UHI) effect is a phenomenon directly linked to rapid urbanization, characterized by the accumulation of heat in urban areas due to human activities and the extensive use of heat-absorbing materials. This UHI effect alters energy and material flows within urban ecological systems, affecting their structure and function and leading to a range of environmental impacts on urban climate, hydrological processes, soil properties, atmospheric conditions, ecosystem dynamics, and human health [1]. Recent studies have shown that the combined effects of UHI and global warming significantly amplify the increase in urban temperature, contributing to heightened heat stress and a corresponding rise in heat-related mortality, particularly in densely populated urban areas [2]. Conventionally used pavement materials in urban settings, such as concrete and asphalt mixtures, have relatively higher solar energy absorption and also possess a higher heat storage capacity that allows them to retain massive heat in daytime and release the heat back into the atmosphere at night [3].

In parallel, as a longstanding challenge in pavement and urban infrastructure design, stormwater runoff has become increasingly critical under rapid urbanization, where the natural hydrological processes are disrupted. The widespread expansion of impervious surfaces, such as pavements and rooftops, limits rainwater infiltration and evapotranspiration, thereby significantly increasing surface runoff, particularly during storm events [4,5]. This altered runoff regime not only amplifies flood risks but also facilitates the transport of pollutants and sediments, leading to contamination of surface water quality. Previous studies have shown that pollutant concentrations are positively correlated with rainfall intensity, further compounding water quality concerns during heavy precipitation events [6]. Moreover, these challenges are being intensified by climate change, which is characterized by rising temperatures, shifting precipitation patterns, and an increased atmospheric moisture capacity, ultimately contributing to more frequent and severe hydrological extremes [7].

Stormwater management in urban areas conventionally relies on centralized collection systems that convey both stormwater and wastewater through pipe networks to nearby water bodies or sewer systems [8]. However, rapid urbanization has led to a substantial increase in impervious surfaces, resulting in greater volumes of runoff and reduced infiltration capacity. Coupled with changing precipitation patterns and the growing frequency of extreme storm events, these systems are increasingly operating beyond their design capacity and are often undersized. As a result, there is a pressing need to expand or upgrade existing infrastructure, which is typically labor-intensive, costly, and disruptive [9]. To address these challenges, decentralized stormwater management strategies, including low-impact development (LID), green infrastructure (GI), sustainable urban drainage systems (SUDS), and the sponge city program (SCP), have been increasingly adopted worldwide. Despite differences in terminology and regional implementation, these approaches have a common principle: promoting infiltration, retention, and evapotranspiration processes at or near the source to mitigate runoff before it enters conventional drainage networks, thereby reducing the volume of water conveyed to centralized drainage systems [10].

Adding to the thermal benefits they provide to urban setups, conventional permeable pavements (PP) have been a key component of LID strategies and are widely applied in urban infrastructure such as roadways and parking facilities [11]. These systems typically consist of surface block pavers underlain by granular base and subbase layers that facilitate the infiltration of stormwater into the subgrade, while also allowing lateral drainage and surface-level pollutant filtration [12]. The storage capacity within these underlying layers plays a critical role in system performance and overall drainage design. Increased storage capacity is particularly beneficial, as it enables the retention of runoff from adjacent impervious surfaces, thereby reducing surface flooding risk, attenuating peak flow rates, and enhancing infiltration processes [13].

Permeable pavements are susceptible to clogging, which significantly reduces their effectiveness in stormwater management. Clogging typically occurs due to the accumulation of debris and fine particles both on the surface and within the pore structure, leading to a decline in infiltration capacity. This would therefore require regular maintenance methods such as pressure washing or vacuum sweeping, which may be required multiple times per year depending on-site conditions and climatic factors [14]. These maintenance demands reduce the long-term economic viability and operational efficiency of permeable pavement systems. Hence, addressing this drawback will improve not just the functionality of permeable pavement blocks in urban scenarios but also their sustainability in reducing the use of resources in their maintenance.

Recent advancements in permeable pavement technology have focused on achieving both high mechanical strength and adequate hydraulic performance. One approach involves minimizing tortuosity, which is defined as the deviation of the fluid flow path from a straight line within a porous medium to enhance drainage efficiency. By reducing tortuosity, high-strength permeable pavements can improve infiltration capacity while mitigating the clogging issues commonly observed in conventional pervious concrete. In this context, a novel permeable pavement system with a low-tortuosity pore structure was first reported by Kia et al. [14]. This low-tortuosity permeable pavement (LTPP) system, coined as clogging-resistant pavement (CRP) or Kiacrete, can be cast in situ and demonstrates both high permeability and improved structural strength, while also exhibiting reduced susceptibility to clogging. Their results indicated improvements in splitting tensile strength and durability, suggesting enhanced resistance to degradation [15]. As such, it has been proposed as an innovative solution for mitigating urban flooding and supporting sustainable urban development. Building upon this concept, further enhancements to the system were made by incorporating monofilament polypropylene microfibers into the clogging-resistant concrete [16], improving the tensile properties and degradation resistance of the concrete.

In addition to calibrating the pore structure of the pavement itself, the selection of aggregate materials can influence the multifunctional performance of pavements. Bottom ash (BA) is an industrial solid by-product generated during coal combustion for power production and is commonly collected from the bottom of coal-fired furnaces [17]. BA has attracted attention as a recycled aggregate because of its porous texture, relatively low density, rough particle shape, and high-water absorption capacity. These characteristics suggest its potential benefit to permeable pavement applications, where infiltration behavior and thermal response are indicators of its functionality [18]. Previous studies have reported the use of coal BA in pavement and pervious concrete materials, indicating its potential contribution to permeability-related performance while supporting the recycling of industrial by-products [19,20]. However, the field-level thermal and hydraulic behavior of permeable pavement systems incorporating bottom ash fine aggregate remains insufficiently investigated, particularly in combination with newer technology such as low-tortuosity flow paths.

Beyond hydraulic performance and fine aggregate selection, the thermal response of permeable pavement is also affected by pore geometry and internal void configuration. Thermal conductivity is a critical property of concrete because it governs heat transfer through conduction and influences surface-temperature dynamics. Previous studies have shown that porosity and tortuosity affect effective thermal conductivity by changing heat-transfer pathways in porous cement-based materials [21]. In addition, void spaces beneath or within structural systems can reduce heat transfer in a manner similar to air-cavity systems used in building envelopes, where air layers act as thermal resistance because air has lower thermal conductivity than solid materials [22].

Despite these advances, previous studies have generally addressed low-tortuosity flow paths, bottom ash aggregate, and subsurface void or storage space as separate design or material concepts. Low-tortuosity pavement studies have mainly focused on permeability, clogging resistance, and mechanical performance [15,16], while bottom ash studies have primarily examined its feasibility as a recycled aggregate in pavement or pervious concrete materials [19,20]. However, the field-level thermal and hydraulic behavior of a precast permeable pavement system that integrates bottom ash fine aggregate, direct vertical infiltration pathways, and modular subsurface storage has not been sufficiently documented. In particular, limited information is available on whether such an integrated system can simultaneously reduce surface-temperature accumulation, promote infiltration and temporary storage, and maintain field stability under vehicle loading.

Therefore, this study combines bottom ash fine aggregate, low-tortuosity infiltration pathways and integrated subsurface storage to improve the thermal regulation and hydraulic functionality of permeable pavement compared with conventional permeable pavement. Accordingly, prototypes were assembled to consist of an LTPP system integrating a bottom ash-based surface block, direct vertical infiltration openings and a modular subsurface storage unit. The proposed system was compared with conventional permeable pavement using pilot-level field tests in terms of representative surface-temperature response before and after water spraying, visible ponding and surface runoff behavior under controlled water application, theoretical storage capacity under field test and observed rainfall scenarios and response after vehicle loading.

## 2. Materials and Methods

### 2.1. Mix Design with Bottom Ash

The mix design of the LTPP blocks used in this study was adopted from the experimental work of Kim et al. [23], as summarized in Table 1. In the referenced study, coal bottom ash, specifically generated from power production, was utilized as a sustainable partial replacement for natural fine aggregate. Compared to natural sand, BA is lightweight, porous, and absorbs more water [24]. The properties of the unprocessed BA used in this study are presented in Table 2. However, its direct use as a fine aggregate or cementitious substitute would require additional crushing or grinding. Previous studies have reported that grinding bottom ash can also increase its effective surface area and water absorption capacity [25], thereby enhancing its benefits to the LTPP system.

To compensate for the associated reduction in mechanical strength with BA as partial aggregate replacements, styrene-butadiene rubber (SBR) latex was incorporated into the mixture. The use of latex-modified concrete has been widely reported to enhance mechanical performance and durability, particularly in mixtures containing recycled materials [26,27]. The proposed mixture was developed to satisfy key performance requirements for permeable pavement materials, including compressive strength, flexural strength, abrasion resistance, slip resistance, freeze–thaw durability and permeability. The results are summarized in Table 3.

### 2.2. Integrated Block Design

#### 2.2.1. Infiltration Block

**Figure 1:**
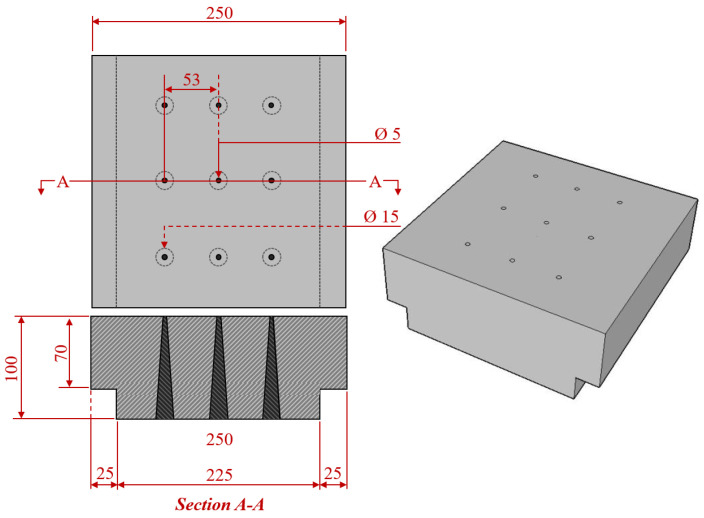
Plan and cross-sectional drawings showing hole arrangement and spacing.

The direct infiltration block of the rainwater infiltration-storage system was designed with dimensions of 250 × 250 mm and a thickness of 100 mm. As illustrated in Figure 1, the block contains nine uniformly distributed surface openings with a diameter of 5 mm. These openings extend through the 100 mm-thick upper block and provide direct vertical pathways from the pavement surface to the underlying storage unit. Based on the 250 × 250 mm dimensions, the nine 5 mm-diameter surface openings provide a total surface opening area of 176.7 mm^2^, corresponding to an opening-area ratio of approximately 0.28%.

The principal infiltration path of the upper block was characterized using a geometry-based tortuosity index:

(1) $$T_{g}=\frac{L_{p}}{H}\;$$

where $T_{g}$ is the geometry-based tortuosity index, $L_{p}$ is the centerline length of the designed through-opening, and H is the vertical block thickness. For the straight vertical openings shown in Figure 1, $L_{p}$ is approximately equal to H; therefore, $T_{g}$ is approximately 1.0 for the primary through-opening. This value, however, should be interpreted as a design-level tortuosity index of the openings rather than the entire pore-network tortuosity of the system. Previous studies have shown that the tortuosity of conventional permeable pavement materials is commonly evaluated using X-ray CT or image-based pore analysis, and reported values can be substantially higher because water passes through irregular, interconnected, and constricted pore networks. For conventional porous pavement specimens based on X-ray tomography and image analysis, Sansalone et al. reported tortuosity values of 2.89–5.91 [33]. Computed tomography has been used to estimate the tortuosity of more conventional permeable concrete, emphasizing that pore characteristics such as connectivity, pore size, and tortuosity must be considered altogether when interpreting permeability [34]. Compared with these irregular pore-network systems, the present infiltration block was designed to provide direct vertical through-openings, thereby reducing the principal flow-path complexity at the block scale. However, since pore-network tortuosity was not directly measured in this study, the comparison is used only to clarify the design rationale rather than to claim a directly measured material tortuosity.

The opening configuration was adopted from the design study by Kim et al. [23], where acrylic specimens were used to evaluate opening diameters of 3, 5.5, 7, and 10 mm and opening-area ratios of approximately 0.12%, 0.25%, and 0.49% before determining the final infiltration-block geometry. In contrast, the conventional permeable pavement section used in the field study relied on the existing permeable block surface, joints, and bedding layer for water movement. Furthermore, Figure 1 shows the block has been designed with opposite notches to fit with the lower infiltration-storage block, thereby ensuring stability and resistance to displacement or settlement under loading conditions.

#### 2.2.2. Storage Block

**Figure 2:**
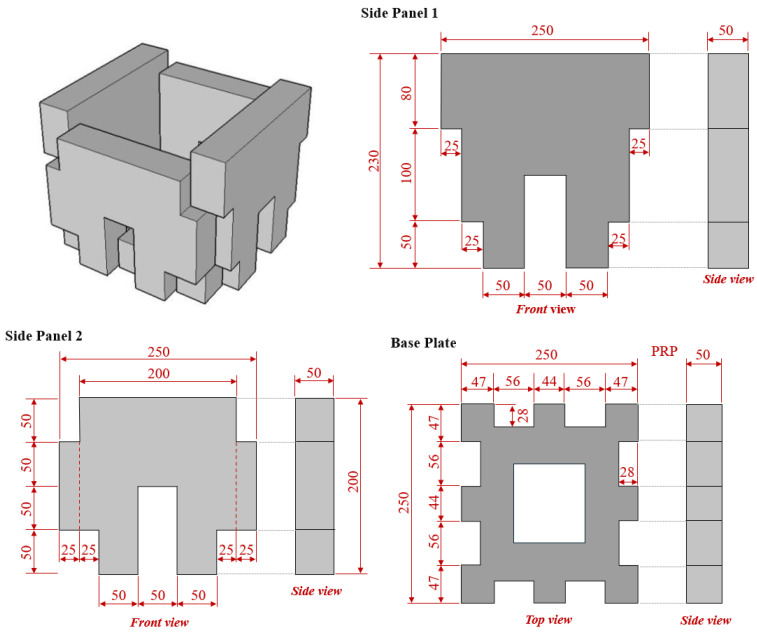
Design and configuration of the lower block base plate and side panels.

The lower storage block was designed to provide a theoretical water storage capacity of approximately 104 L/m^2^, corresponding to approximately 104 kg of water per 1 m^2^ and an equivalent storage depth of 104 mm [23]. Figure 2 shows the system consisting of a base plate and two side panels, which are fabricated separately to facilitate ease of manufacturing and assembly. Additionally, corresponding notches have been incorporated at the edges of the blocks to facilitate interlocking between adjacent units and ensure stable joint connections. Void spaces can be found on the lower portion of the side panels, as illustrated in Figure 2, to allow for water movement and temporary storage within the system. The assembled configuration provides a stable base for the upper block while maintaining sufficient internal storage volume and allowing hydraulic connectivity and drainage ways to future expansions of the system.

#### 2.2.3. Interlocking Design

**Figure 3:**
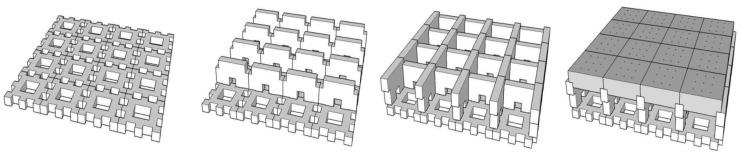
Assembly sequence of the rainwater infiltration-storage block.

The developed rainwater infiltration-storage block was designed as an integrated system to enhance both permeability and storage capacity. The components were configured to interlock through fitted lap edge joints as seen in Figure 3, enabling dry precast and modular assembly, which reduces construction time [35] while delivering adequate axial load-bearing capacity under repeated vehicular loading, along with improved resistance to settlement and lateral displacement.

### 2.3. Field Demonstration

To evaluate field performance, the pilot installation of prototypes was conducted at a wastewater treatment facility located in Boseong-ri, Useong-myeon, Gongju-si, Chungcheongnam-do, South Korea. The selected site, typically used for vehicle parking, was previously paved with conventional PP.

**Figure 4:**
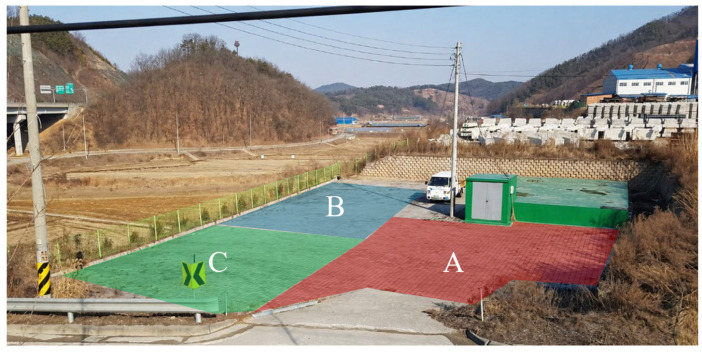
Division of site for field validation.

As shown in Figure 4, the site was divided into three test sections. Section A consisted of the pre-existing PP blocks. Section B was designated for the installation of the proposed LTPP infiltration-storage system. Section C comprised the same surface pavers installed directly over a sand bedding layer without the underlying storage structure. Section C was specifically configured to provide a comparative basis for evaluating resistance to displacement and settlement, particularly with respect to the structural performance of the dry jointed connection system used in the infiltration-storage configuration. In addition, this section enabled the assessment of the standalone performance of LTPP with bottom ash under stormwater conditions, isolating the influence of the storage layer. This provides a preliminary basis for assessing the feasibility of using bottom ash low-tortuosity blocks independently when environmental, cost, or construction constraints render the use of storage layers impractical.

**Figure 5:**
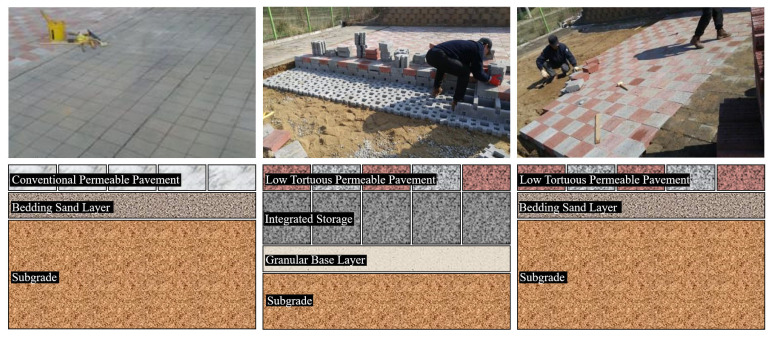
Configurations of permeable pavements used for field validation.

Figure 5 illustrates the three pavement configurations implemented for field validation. The conventional permeable pavement system consists of surface permeable blocks that had been installed over a bedding sand layer and subgrade. The proposed LTPP with integrated storage incorporates an additional subsurface storage layer supported by a granular base, enabling enhanced infiltration and temporary retention of stormwater. In contrast, the LTPP-only system comprises identical surface blocks placed directly over a bedding sand layer without the underlying storage component. All pavement configurations, like the pre-existing PP blocks, were installed with dry joint sand to ensure consistent inter-block filling and surface stability across test sections.

#### Pavement Surface Temperature Measurement

To evaluate the thermal performance of the pavement systems, surface-temperature measurements were conducted using a handheld infrared thermal imaging camera (Testo 882, Testo SE & Co., KGaA, Lenzkirch, Germany) with specifications provided in Table 4.

Measurements were carried out between 15:20 and 17:20 at 30 min intervals. Table 5 summarizes the atmospheric conditions during the testing period. The testing period was characterized by warm air temperature, continuous sunshine, and measurable solar radiation, which provided favorable daytime heating conditions for evaluating the relative surface-temperature response of the LTPP and conventional PP sections [36]. Pavement surface temperature is strongly affected by atmospheric variables such as solar radiation, ambient air temperature, wind speed, and humidity; therefore, these conditions were considered when interpreting the thermal imaging results [37]. However, because the measurements were conducted under field conditions rather than a fully controlled thermal environment, the results were interpreted as a relative comparison between adjacent pavement sections exposed to the same atmospheric conditions.

**Figure 6:**
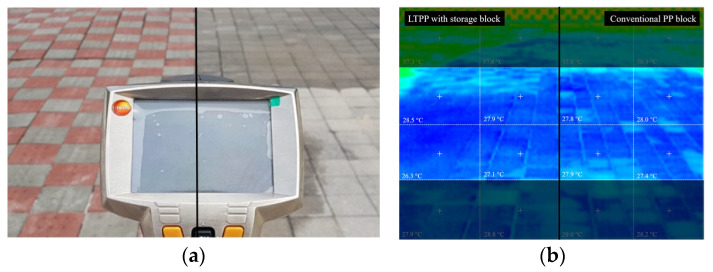
Thermal imaging measurement: (**a**) delineation of adjacent pavement sections for comparative temperature analysis; (**b**) representative ROI selection from the thermographic image for surface-temperature extraction.

Dry-condition surface temperatures were first measured, after which the pavement surface was wetted using a sprinkler truck to simulate rainfall conditions. Thermal imaging was subsequently conducted by distinguishing between the LTPP infiltration-storage block section and the conventional PP block section, as shown in Figure 6a. For each thermographic image, regions of interest (ROI) were selected within each pavement section, and the same ROI-based temperature extraction procedure was applied to both sections. Because the thermal survey was measured using ROIs on the LTPP and adjacent conventional PP sections, the results should be interpreted as representative temperatures rather than successive measurements.

### 2.4. Functional Performance Evaluation

**Figure 7:**
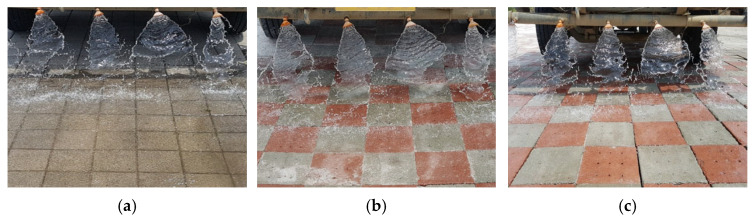
Rainwater permeability test setup: (**a**) PP (Section A); (**b**) LTPP with integrated storage (Section B); (**c**) LTPP-only (Section C).

Simulated rainfall conditions, as shown in Figure 7, were applied to evaluate surface runoff characteristics, ponding behavior, and infiltration response under controlled water application. The water-spraying test was conducted at a simulated rainfall intensity of approximately 15.63 mm/h for 2 h. Multi-gradient rainfall intensities were not applied in this field test; therefore, findings should be interpreted as a qualitative field assessment under a single moderate rainfall condition. Table 6 summarizes the test configurations. This field-based assessment was conducted to confirm whether the pilot-level LTPP prototype could retain its intended infiltration-storage function after initial installation and repeated vehicular loading.

**Figure 8:**
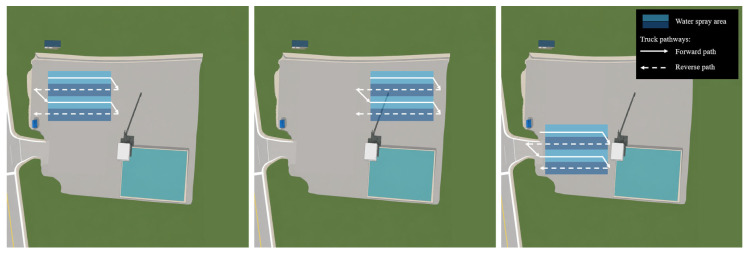
Field-test layout showing the water-spray areas and vehicle-movement paths during rainfall simulation.

A vehicle-loading observation was conducted for each pavement setup using the sprinkler truck, which had an estimated vehicle weight of 5–6.5 tons and an additional water tank capacity of approximately 5 tons, resulting in a total load exceeding 10 tons. The water-spraying truck moved along the designated paths shown in Figure 8, and the shaded region indicates the approximate water-spray area used during the field test. For each 80 m^2^ configuration, the sprinkler truck followed a straight-line movement path for four repeated cycles, with water spraying continued for a total duration of 2 h. The post-loading surface condition of the pavement blocks was then visually examined to assess their field stability. Particular attention was given to observing the response of each pavement configuration to the applied load, including potential displacement, settlement, and surface integrity. Since rut depth and displacement were not quantitatively determined in this pilot-level study, any observation should not be interpreted as a full mechanical-performance evaluation.

### 2.5. Theoretical Assessment of Storage Capacity Under Observed Rainfall

**Figure 9:**
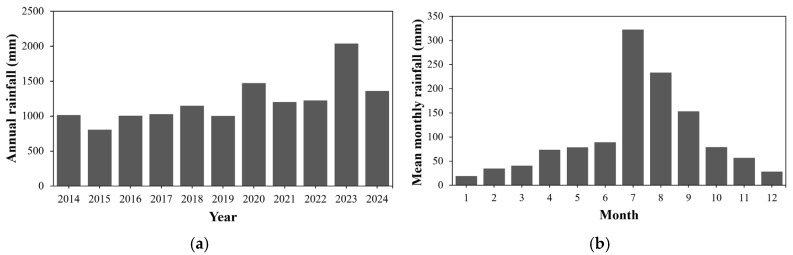
Cumulative rainfall data [38] in Useong-myeon, Gongju: (**a**) annual; (**b**) mean monthly rainfall.

The pilot installation was located in Useong-myeon, Gongju, where the annual rainfall from 2014 to 2024 ranged from 804.5 mm in 2015 to 2038.0 mm in 2023, as illustrated in Figure 9a. This confirms that the field site experiences rainfall conditions in which temporary storage and delayed runoff release are important for pavement performance. As shown in Figure 9b, the mean monthly rainfall was strongly concentrated during the summer season, with July and August recording the highest values of 322.5 mm and 233.0 mm, respectively. Therefore, the storage response of the LTPP system was theoretically assessed to evaluate its potential to retain and gradually release captured stormwater under rainfall conditions representative of the study area.

To evaluate the potential storage response of the LTPP system, a storage-capacity assessment was conducted using the observed rainfall data. For a rainfall event with total rainfall depth *P*, the storage utilization ratio was calculated as:

(2) $$U_{s}=\frac{P}{S_{d}}\;\times 100\%$$

where $S_{d}$ is the equivalent storage depth of the LTPP system (mm). This ratio represents the percentage of the theoretical storage depth that could be filled by the observed rainfall event.

For a rainfall intensity I, the time required to fill the storage depth under static storage conditions was calculated as:

(3) $$t_{s}=\frac{S_{d}}{I}\;$$

where $t_{s}$ is the theoretical filling time, and I is the rainfall intensity. This calculation estimates the time required to fill the storage volume over the pavement footprint before considering infiltration into the subgrade, lateral drainage, evaporation, or overflow. Therefore, the calculated filling time should be interpreted as a conservative estimation under no-outlet storage conditions.

Delayed-drainage storage behavior was calculated using a 10 min time-step water balance based on the recorded rainfall series. For each step, the stored depth was updated as follows:

(4) $$s_{t}=min[s_{max},\;max(0,\;s_{t-1}+P_{t}-D_{t})]$$

where $s_{t}$ is the stored water depth at time step t, $s_{max}$ is the maximum storage capacity of the LTPP system at 104 mm, $s_{t-1}$ is the stored depth at the previous time step, $P_{t}$ is the rainfall depth during the 10 min interval, and $D_{t}$ is the drainage depth during the same interval. The drainage depth was calculated as:

(5) $$D_{t}=\frac{D_{r}}{6}$$

where $D_{r}$ is the assumed drainage rate in mm/h. Overflow at each time step was calculated as:

(6) $$O_{t}=max(0,\;s_{t-1}+P_{t}-D_{t}-\;s_{max})$$

Cumulative overflow was obtained by summing $O_{t}$ over the rainfall event. Lastly, storage utilization was calculated as the maximum stored depth divided by $s_{max}$ and emptying time was calculated as the final stored depth divided by the assumed drainage rate.

Since the pilot installation did not include direct drainage or outflow measurement, drainage, as summarized in Table 7, was treated as a scenario parameter rather than a measured value. This analysis therefore provides an estimation of the storage behavior of the LTPP system under observed rainfall conditions.

**Figure 10:**
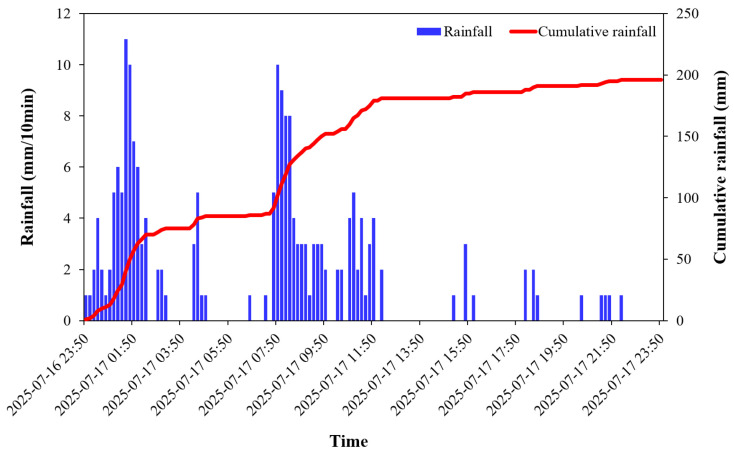
Rainfall conditions in a 24 h sample on 16–17 July 2025.

As shown in Figure 10, intermittent rainfall data was obtained from the Geum River Flood Control Office online public repository for the Gongju-si (Geumgang Bridge) observation station on 16–17 July 2025, reaching a total cumulative rainfall depth of 196 mm. This rainfall data was used to calculate the storage response of the LTPP system.

## 3. Results

### 3.1. Thermal Performance

**Figure 11:**
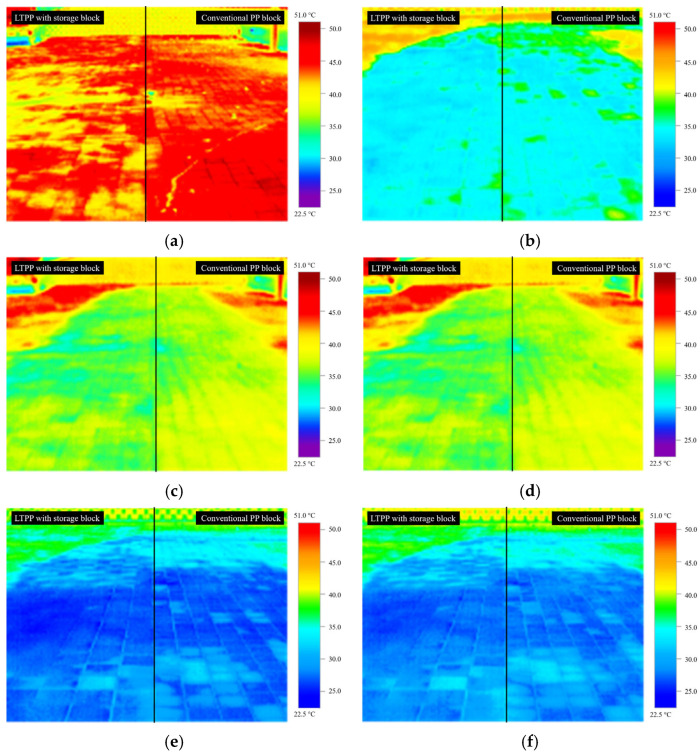
Thermographic images showing the temporal evolution of surface temperature for the LTPP system (**left**) and conventional PP (**right**): (**a**) dry condition; (**b**) immediately after water application; (**c**) 30 min; (**d**) 60 min; (**e**) 90 min; and (**f**) 120 min.

Figure 11 shows the temporal evolution of pavement surface temperature for the LTPP system with integrated storage and the adjacent conventional PP section. Under dry conditions, a larger proportion of the LTPP section exhibited comparatively lower temperature regions, suggesting reduced heat accumulation relative to the conventional PP section. This reduction may be attributed to the combined effects of the drainage openings, internal void/storage configuration, and porous characteristics of the bottom ash fine aggregate. The drainage openings act as air gaps within the concrete block, thereby reducing the thermal conductivity of the block [40]. Under dry conditions, these pores are filled with air; consequently, the air-filled pores interrupt conductive heat-transfer pathways and reduce the effective thermal conductivity of the material [41].

Immediately after water spraying, both pavement sections showed a rapid decrease in surface temperature due to wetting and evaporative cooling. Figure 11b shows localized cooler regions indicative of surface ponding of uninfiltrated water. These localized cooler regions dissipated rapidly through evaporation, as observed in Figure 11c,d. Figure 11e represents the lowest surface temperatures recorded for both configurations; however, the conventional PP section exhibited a faster reheating tendency, as shown in Figure 11f.

**Figure 12:**
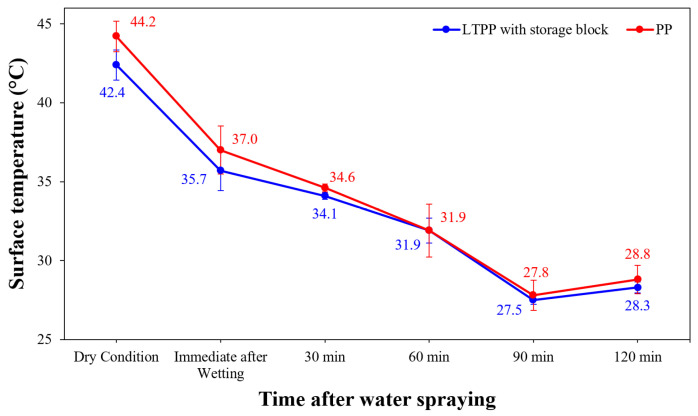
Comparison of surface-temperature evolution for LTPP with integrated storage and conventional PP.

Figure 12 presents the surface-temperature evolution of the LTPP system and conventional PP based on four ROI readings from each pavement section. Under dry conditions, the LTPP system showed a mean surface temperature of 42.4 ± 1.0 °C, whereas conventional PP showed 44.2 ± 1.0 °C, corresponding to an improved initial temperature of 4.07%. This indicates an inherent thermal advantage of the LTPP system. The LTPP system with integrated storage consistently maintained lower surface temperatures throughout the monitoring period. This behavior can be attributed to a dual-level moisture retention mechanism, which promotes internal evaporative cooling by diverting thermal energy toward phase-change processes [42]. This mechanism is supported not only by the increased porosity of the upper block and the integrated storage layer beneath the pavement, where air and infiltrated water can be temporarily retained, but also by the porous and water-absorptive characteristics of the bottom ash fine aggregate, which may enhance moisture retention within the pavement matrix and sustain evaporative cooling after wetting. This aligns with a key principle in porous pavement design for urban thermal regulation, where increased water storage capacity contributes to prolonged cooling performance.

The evaporation-induced cooling effect becomes more evident during the reheating phase between 90 and 120 min, where the LTPP system exhibited a slower rate of temperature increase compared with conventional PP. Although the temperature readings converged at approximately 60 min, the subsequent temperature rise remained more controlled, with a heat gain of 2.91% compared with 3.60% for the PP system. In addition, water application induces convective heat exchange between the cooler infiltrated water and the heat stored within the pavement matrix [43], further contributing to the observed cooling behavior. Overall, the combined effects of higher porosity of the infiltration block and its recycled aggregate, reduced tortuosity, and integrated subsurface storage enhanced both moisture retention and heat-transfer pathways, resulting in a more thermally stable pavement system characterized by sustained cooling and delayed temperature recovery. These results suggest that the LTPP system can reduce surface heat accumulation under the tested field conditions. Although this does not directly verify large-scale UHI effects, it represents a preliminary pavement-scale contribution to UHI mitigation by improving surface-temperature regulation.

### 3.2. Infiltration and Storage Evaluation

**Figure 13:**
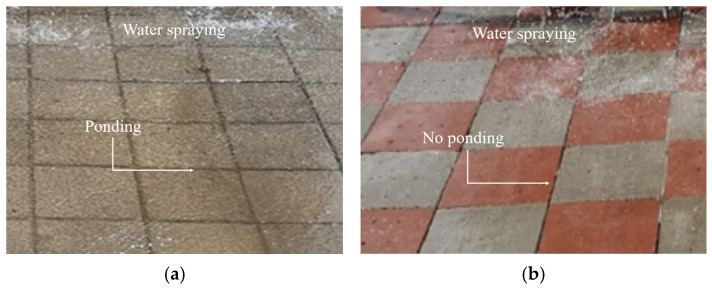
Observations of ponding during water spraying: (**a**) PP; (**b**) LTPP.

For the conventional permeable concrete pavement, both ponding and surface runoff were observed, as seen in Figure 13a. This behavior is attributed to the accumulation of foreign materials, such as fine dust and soil, on the pavement surface over long-term service, which likely clogged the pore structure of the blocks. This ponding was also evident in the thermal imaging results discussed in Section 3.1, where these accumulations were read as the cooler regions on the surface of the PP configuration right after water spraying. Although water was able to infiltrate through the block surface and inter-block joints, the absence of an underlying storage mechanism limited its retention capacity, resulting in surface runoff once saturation was reached. Ponding prevention should be of importance to permeable pavement design as it is correlated with the progression of roadway deterioration [44,45].

Given that the conventional permeable pavement at the test site had experienced prolonged use and pre-existing wear, a comparative configuration was established to evaluate the performance of the LTPP blocks under similar conditions but without the influence of a storage layer. Accordingly, a test section was constructed using the same LTPP surface blocks placed over a bedding sand layer directly on the subgrade, replicating the conventional pavement conditions. In this configuration, surface runoff was still observed. Similar to the conventional section, rainwater infiltrated through both the block surface and joints; however, due to the absence of subsurface storage, runoff occurred after a delay compared to PP. Notably, ponding was not observed, as seen in Figure 13b, in the sections with the LTPP blocks, which may be attributed to the clogging-resistant nature of the upper blocks. At the section where the complete rainwater infiltration-storage system was installed, no visible surface ponding or runoff was observed during the 2 h water-spraying test. Water was observed to enter through the block surface and interlocking joints and to move toward the subsurface storage layer. The applied water volume during the test was approximately 2.5 m^3^, whereas the theoretical storage capacity over the same 80 m^2^ test area was approximately 8.32 m^3^, based on the storage capacity of 104 L/m^2^. The applied water volume therefore corresponded to 30.1% of the theoretical storage capacity of the test area. This storage margin is consistent with the absence of visible ponding or surface runoff under the selected water-spraying condition.

### 3.3. Preliminary Visual Observations Under Vehicle Loading

**Figure 14:**
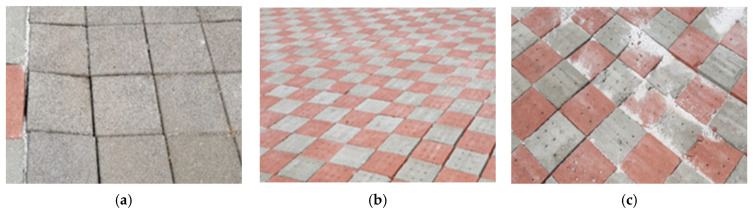
Preliminary visual observation after vehicle-load application, including the truck wheel path and observed displacement locations: (**a**) conventional PP; (**b**) LTPP with integrated storage; (**c**) LTPP-only.

Observations of the PPs under vehicle loading were localized settlement and displacement, particularly in areas adjacent to the newly installed low-tortuosity permeable pavement blocks, as shown in Figure 14a. This behavior is attributed to disturbance of the underlying sand bedding during installation near the interface with the newly constructed sections, combined with pre-existing erosion, resulting in differential settlement. In contrast, blocks located toward the central region exhibited relatively less visible displacement; however, instability between the blocks was still evident, as indicated by audible responses under the wheel load.

In contrast, the LTPP system with integrated storage exhibited minimal surface disturbance under vehicular loading, as seen in Figure 14b. This improved performance is attributed to the interlocking connection between the upper infiltration blocks and the underlying storage units, as well as the interconnection among the storage blocks themselves. This configuration facilitates more uniform load transfer across the pavement system, reducing localized stress concentrations. In addition, the dry jointing mechanism enhances frictional resistance at the block interfaces, contributing to improved resistance against displacement and settlement under repeated loading.

Since the conventional PP was already being used on the selected site prior to field testing, it was also necessary to have a comparison of how newer blocks would perform under repeated loading, such as vehicular loads, in a setup that does not utilize the interlock of the infiltration-storage system. There was rutting failure occurring in a T-shape pattern along the path of vehicle loads, as seen in Figure 14c. The relatively thin sand layer beneath the blocks as well could potentially have caused this failure. Increasing bedding thickness and minimizing joint widths could improve the interlock [46] between the LTPP blocks, should they be utilized independently. The observed displacement patterns for both sections with PP and LTPP without the integrated storage may have been influenced by the truck wheel path, local bedding conditions, and section boundary effects. However, because rut depth, settlement, lateral displacement, and repeated load response were not quantitatively measured, the findings of this preliminary field test should be interpreted as qualitative field observations.

### 3.4. Theoretical Storage Performance and Drainage Calculations

In addition to the controlled water-spraying test, rainfall-storage calculations were performed using observed rainfall records to examine the storage demand of the LTPP system under recorded rainfall accumulation scenarios. For 1 m^2^ of the block system, the internal storage capacity was designed to store a maximum of 104 L, corresponding to an equivalent storage depth of 104 mm. A total of 1408 block units were installed within 88 m^2^ of the field site, providing an overall storage capacity of approximately 9.15 m^3^, or 9152 L, of rainfall storage. Based on the simulated rainfall intensity of 15.63 mm/h, the theoretical static-storage duration was estimated using two calculation bases. First, the unit-area storage duration was calculated using the nominal storage depth of 104 mm; second, the field-test-area storage duration was calculated by comparing the total installed storage volume of 9152 L with the simulated rainfall input over the 80 m^2^ test area:

$$t_{s}\left\{\begin{array}{c} \frac{104\;L}{15.63\frac{mm}{h}}=6.65\;h\\ \;\frac{9152\;L}{\left(15.63\frac{mm}{h}\right)\left(80\;m^{2}\right)}=7.32\;h \end{array}\right.$$

Thus, the storage duration was estimated to be approximately 6.65 to 7.32 h under static-storage conditions, before considering infiltration into the subgrade, lateral drainage, evaporation, or overflow. This indicates that the storage capacity substantially exceeded the simulated rainfall input during the field test, potentially buffering runoff. Therefore, the observed absence of ponding or runoff can be attributed to the combined effects of direct infiltration through the low-tortuosity upper block, moisture retention associated with the bottom ash fine aggregate, and temporary bulk storage provided by the lower storage block.

However, the simulated rainfall input generated by the water-spraying test was only 15.63 mm/h, corresponding to approximately 30% of the 104 mm unit storage depth for the duration it was conducted. Therefore, although this event was useful for confirming short-term wetting, infiltration, and post-rainfall thermal response, it is not hydraulically severe enough to fully evaluate the storage capacity of the system. To further assess storage demand under a more critical rainfall condition, an additional scenario analysis was conducted using historical rainfall data from the Gongju-si (Geumgang Bridge) station, as summarized in Table 8. In this scenario under a static-storage assumption, the 24 h cumulative rainfall depth reached 196 mm, corresponding to 188.5% of the available storage depth. The historical rainfall scenario further supports the theoretical static-storage duration estimated from the simulated rainfall intensity. Consistently, the historical rainfall data showed that the maximum 6 h rainfall depth was 85 mm, corresponding to 81.73% of the storage depth, while the 9 h rainfall depth reached 137 mm, exceeding the available storage depth. This indicates that the storage layer would be sufficient for short- to medium-duration rainfall accumulation, while overflow risk would be associated mainly with prolonged rainfall exceeding the available storage depth.

Table 9 presents a summary of the delayed-drainage scenario analysis. Under the static-storage condition, the 196 mm rainfall event exceeded the available 104 mm storage depth, causing the storage layer to reach full capacity and resulting in a cumulative overflow depth of 92.0 mm. When slower-ranged drainages were considered, the system still reached full storage capacity; however, increasing the drainage rate reduced the cumulative overflow depth from 52.0 mm at 2 mm/h to 14.5 mm at 5 mm/h. At the design-threshold drainage rate of 15 mm/h [39], the system did not reach full storage capacity, with a maximum stored depth of 34.5 mm and no predicted overflow. At 30 mm/h, the maximum stored depth further decreased to 15.0 mm, indicating rapid drainage relative to rainfall input. This suggests that the integrated storage layer can buffer rainfall effectively when combined with sufficient subgrade infiltration or outlet drainage. Therefore, the hydraulic performance of the LTPP system should be interpreted as a combined function of storage capacity, rainfall duration, and effective drainage rate, rather than storage capacity alone.

## 4. Discussion

This section discusses the contribution of the bottom ash fine aggregate and the configuration and design of the pavement system to the observed thermal, hydraulic, and storage-related performance of the LTPP system.

### 4.1. Bottom Ash Characteristics and Potential Contribution

**Figure 15:**
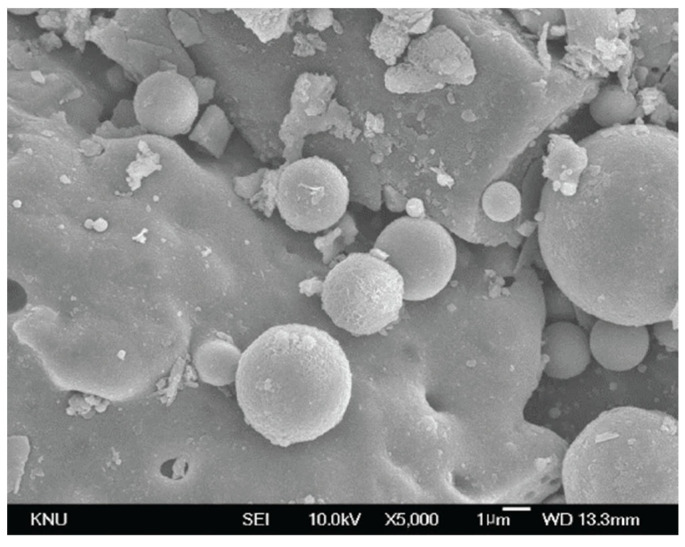
SEM images of the bottom ash fine aggregate used in the mix design.

To further assess the suitability of BA as a fine aggregate replacement in the permeable pavement system, SEM imaging was conducted on the raw BA fine aggregate to observe its particle morphology and surface characteristics. Prior to observation, the raw BA particles were dried, mounted on carbon tape, and conductively coated. The SEM image was obtained in secondary electron imaging mode using a MIRA LMS microscope at a magnification of ×5000, an accelerating voltage of 10.0 kV, and a working distance of 13.3 mm. As shown in Figure 15, the ash-based fine aggregate exhibited a mixed morphology, including smooth spherical particles and irregular rough-surfaced fragments.

The spherical particles are consistent with glassy ash microspheres formed during combustion, whereas the irregular and angular fragments are more characteristic of BA [47,48]. This mixed morphology suggests that the material may provide both improved workability, associated with the spherical particles, and enhanced water absorption or moisture retention, associated with the rough and porous particles [47,48,49]. These characteristics are particularly important for thermal regulation because retained moisture can support evaporative cooling after wetting.

Previous studies have reported that the inclusion of BA in pervious or porous concrete can influence thermal behavior because of its porous internal structure and aggregate characteristics. Ngohpok et al. [19] reported that pervious concrete containing bottom ash aggregate showed different thermal conductivity behavior depending on aggregate type and mixture characteristics. Jeong et al. [50] further showed that the thermal conductivity of coal-bottom-ash porous concrete varied with compaction level and aggregate size. The porous structure of bottom ash may also influence heat transfer within the pavement block [41,51]. In a dry, air-filled state, internal pores can act as insulating voids, thereby reducing effective thermal conductivity. This dual behavior suggests that BA may support both dry-condition thermal insulation and wet-condition evaporative cooling. These findings suggest that BA-containing porous materials may affect heat transfer within pavement systems through changes in pore structure, air voids, and moisture retention.

In the current study, the delayed surface-temperature recovery observed after water spraying may therefore be partly associated with the water-retentive characteristics of the BA fine aggregate, together with the storage-enabled configuration of the LTPP block and integrated storage layer. In a dry state, the porous structure may reduce heat transfer through air-filled voids, whereas in a wetted state, retained water may contribute to evaporative heat removal. However, because internal moisture distribution, evaporation rate, and heat flux were not directly measured, this mechanism should be interpreted as a plausible explanation supported by previous studies rather than as direct experimental proof.

### 4.2. Design and Economic Implications of the LTPP System

**Figure 16:**
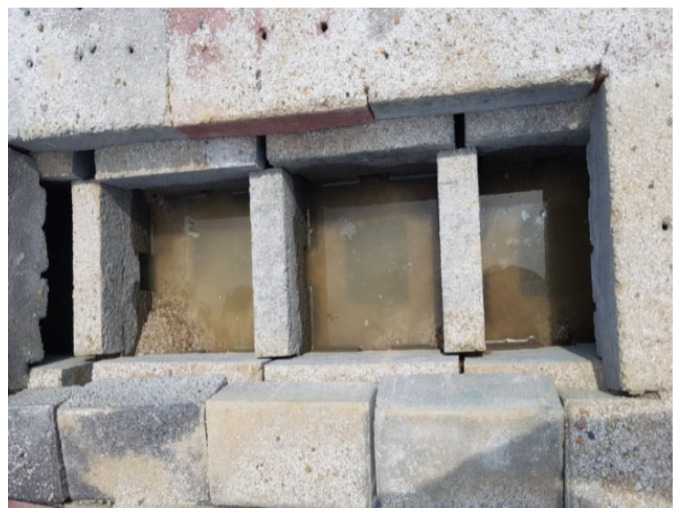
Rainwater storage condition of the storage block.

Inspection confirmed that water was effectively retained within the lower portion of the infiltration-storage system. The simulated rainfall test provided pilot-scale visual confirmation that water entered the storage block and remained within the storage space after water spraying, as shown in Figure 16. However, the test did not include direct measurements of inflow, outflow, stored water volume, overflow, storage drawdown, or infiltration into the underlying soil. Therefore, field observations should be interpreted as qualitative evidence of water entry and temporary storage, not as quantitative verification of hydraulic performance. To supplement this limitation, the storage-capacity and delayed-drainage analyses were conducted as theoretical, scenario-based assessments using the available storage depth, recorded rainfall data, and assumed effective drainage rates.

The theoretical storage analysis was conducted to estimate the potential storage response of the LTPP system under the tested and recorded rainfall conditions. Under the simulated rainfall intensity of 15.63 mm/h, the static-storage duration was estimated as 6.65–7.32 h, indicating that the available storage depth could accommodate the applied water-spraying condition under static-storage assumptions. Historical rainfall analysis further contextualized the storage capacity. The maximum rolling 6 h rainfall depth was 85 mm, corresponding to 81.73% of the available 104 mm storage depth, whereas longer-duration rainfall exceeded the storage capacity. This suggests that the LTPP system may provide temporary buffering for short- to medium-duration rainfall events, while prolonged rainfall would require effective drainage or outlets. However, these estimations should not be interpreted as a measured storage duration because direct inflow, outflow, stored water volume, overflow, and storage drawdown were not measured during the field test. Therefore, future laboratory and field evaluations should include direct hydraulic measurements and design improvements that promote controlled drainage.

Furthermore, the influence of albedo on surface temperature should also be considered, as previous studies have demonstrated that pavement color and surface morphology can significantly affect thermal performance. In particular, red jagged pavers have been reported to contribute to lower pavement temperatures compared to other color-texture configurations [52]. Although both PP and LTPP systems in this study utilized similar light-gray pavers, the presence of red blocks for the latter may have contributed to localized reductions in surface temperature. However, it is important to note that while the red jagged paver surfaces can enhance thermal performance, they may not be suitable for applications involving significant pedestrian traffic due to reduced surface comfort and safety. In this context, the selection of moderately rough or textured pavers represents a practical compromise, offering potential thermal benefits without compromising usability. These results suggest that surface characteristics such as color and texture can also play a secondary but non-negligible role in influencing pavement thermal behavior aside from esthetic purposes.

A preliminary production-cost comparison was also considered to assess practical applicability. Based on the available product-level unit-cost data, the proposed LTPP system requires both an upper permeable block and a lower storage block. The production cost of the upper block was estimated as 22,852 KRW/m^2^ for the gray type and 23,404 KRW/m^2^ for the colored type, while the production cost of the lower storage block, including the side-wall block components, was estimated as 13,197 KRW/m^2^. Therefore, the combined product-level cost of the LTPP system was estimated as 36,049 KRW/m^2^ for the gray-block configuration and 36,601 KRW/m^2^ for the colored-block configuration. For comparison, publicly available product prices for conventional permeable concrete blocks were 18,600 KRW/m^2^ (Dongil Concrete, Gimhae, Republic of Korea), 27,000 KRW/m^2^ (Sunroad, Ansan, Republic of Korea), and 27,500 KRW/m^2^ (Daeiltec, Eumseong, Republic of Korea). These values indicate that the proposed LTPP system has a higher initial product-level cost than conventional permeable pavement. However, the estimation is limited to product-level production costs and does not include installation, transportation, drainage connection, maintenance, service life, or life-cycle benefits.

Although the initial production cost of the LTPP system is higher than that of conventional permeable pavement, its integrated storage function may provide additional long-term value when the outlet is applied as a non-potable water reuse system. Stored stormwater can be integrated into existing non-potable water supply systems within buildings, supporting applications such as toilet and urinal flushing, irrigation, vehicle and equipment washing, and fire protection [53]. Hence, the future integration of a drainage outlet into the LTPP system can be designed to convey temporarily stored water to a collection tank or reuse system, enabling the stored runoff to be utilized as an alternative non-potable water source. Such integration can result in substantial reductions in potable water demand, along with potential energy savings compared to conventional non-harvesting systems [54]. By decreasing reliance on a centralized water supply, this approach potentially offers notable environmental benefits and helps alleviate pressure on public water infrastructure.

Additionally, the modular configuration of the system enables localized inspection, maintenance, and replacement by selectively removing individual blocks without disturbing the surrounding pavement. This feature offers a practical advantage for long-term operation, as potential issues such as clogging, reduced storage performance, or structural damage can be addressed efficiently at specific locations, thereby enhancing the sustainability of the pavement system’s service life.

### 4.3. Limitations of the Study

Despite these findings, several limitations should be considered when interpreting findings of this pilot-level field study. First, the thermal imaging survey was conducted on ROIs of the LTPP section and an adjacent conventional PP section; therefore, the temperature profiles represent field-observed measurements rather than statistically replicated results. In addition, as discussed in the previous section, surface color and albedo may also have affected the measured surface temperatures. Therefore, the observed thermal improvement should not be attributed solely to any single factor, such as internal block geometry, bottom ash incorporation, or the subsurface storage layer. Rather, the thermal response should be interpreted as the combined effect of multiple design features that may contribute together in regulating surface temperature under the tested field conditions.

Second, although the water-spraying test confirmed rapid infiltration and temporary subsurface storage, the experimental setup was limited to the use of a single simulated rainfall intensity of 15.63 mm/h for 2 h and visual observations of ponding and runoff. Multi-gradient rainfall intensities were not experimentally verified, and direct measurements of infiltration rate, peak flow rate, runoff reduction, and outlet discharge were not conducted. Therefore, these preliminary hydraulic observations should be interpreted together with the theoretical storage calculations as baseline evaluation rather than as a complete hydrological performance validation. Future studies should employ controlled rainfall simulations with multiple rainfall intensities and direct inflow–outflow monitoring to quantify the hydrological performance of the LTPP system more rigorously.

Third, the role of bottom ash was evaluated based on the adopted mix design, previously reported material performance, and qualitative particle morphology. Because the field comparison did not include a bottom-ash-free LTPP section, the independent contribution of bottom ash to field thermal response or water-retention behavior could not be separated from the effects of direct infiltration openings, surface characteristics, and the storage layer. A recommended extension of this pilot-level field evaluation is a comparison of LTPP blocks with and without BA to quantify the specific influence of BA on water retention, permeability, thermal conductivity, heat accumulation and release, and mechanical durability.

Finally, the effects of vehicle loading on the configurations were based on visual post-loading inspection where rut depth and displacement were not quantitatively monitored. Further instrumented field studies and standardized mechanical tests are warranted to validate load-induced performance of the LTPP system and its components.

## 5. Conclusions

This study presents a pilot-scale field demonstration of a modular, interlocking LTPP system incorporating a BA-containing surface block and an underlying storage structure. The findings demonstrate that combining low-tortuosity pore structures with a storage-enabled interlocking configuration could potentially provide a multifunctional improvement over conventional permeable pavement.

- Compared with conventional PP, the LTPP system reduced surface temperature by 4.07% under dry conditions and by 3.51% immediately after wetting. During the reheating period from 90 to 120 min, the LTPP system showed a smaller temperature increase of 2.91%, compared with 3.60% for conventional PP, indicating improved moisture retention and thermal stability.
- Observations after simulated rainfall on the pilot-level installation of the prototype LTPP system confirm that water infiltrated and was stored in the storage block of the pavement system.
- The LTPP system is designed to store an equivalent depth of 104 mm of water per 1 m^2^. Under the simulated rainfall intensity, the design-based, static-storage duration of the installed setup was estimated as 6.65–7.32 h. The total storage capacity was calculated to be approximately 9.1 tons for an area of 88 m^2^, demonstrating its potential for stormwater management, non-potable water reuse, and localized maintenance.
- Preliminary visual inspection after vehicle-load application showed that visible rutting and displacement were mainly observed in the conventional PP and LTPP-only configurations, whereas, likely due to the interlocking nature, the complete LTPP system with integrated storage showed no obvious surface displacement under the tested condition.

Overall, the findings indicate that the proposed LTPP system has potential as a multifunctional pavement for surface-temperature regulation, temporary stormwater storage and field-level stability. However, the results should be interpreted within the scope of a pilot-scale field demonstration. Further studies are required to quantify infiltration rate, runoff reduction, storage drawdown, outlet discharge, long-term clogging behavior, load-induced deformation, and life-cycle economic performance under controlled laboratory and replicated field conditions.

## Figures and Tables

**Table 1:** Mix design of the LTPP blocks [23].

Cement	Water	Fine Aggregate	Bottom Ash	Coarse Aggregate	Latex
(kg/m^3^)					
450	129.5	855.27	95.03	633.6	100

**Table 2:** Properties of bottom ash.

Particle Size (mm)	Absolute Density (g/cm^3^)	Absorption (%)	Abrasion (%)
<5	3.2	3.15	0.02

**Table 3:** Mechanical performance of the LTPP blocks made according to the configured mix design [23].

Indicator	Standard	Target	Results
Compressive strength (MPa)	KS F 2405 [28]	≥40	42.33
Flexural strength (MPa)	KS F 2408 [29]	≥4.5	5.17
Coefficient of Permeability	KS F 4419 [30]	≥0.5	1.30
Freeze–thaw resistance (%)	KS F 2456 [31]	≥80	87
Slip resistance (BPN)	ASTM C944/C944M [32]	≥68	70

**Table 4:** Thermal imaging camera baseline specifications.

Specification	Description
Model	Testo 882
Resolution	640 × 480 pixels/1.1 mrad (standard lens)
Instantaneous field of view	1.7 mrad (standard lens)
Temperature range	Switchable ranges: −20 to 100 °C; 0 to 350 °C
Accuracy	±2 °C or ±2%

**Table 5:** Meteorological conditions during thermal imaging measurements.

Condition	15:00	16:00	17:00	18:00
Air Temperature (°C)	29	29	29	28
Humidity (%)	44	44	46	49
Windspeed (m/s)	3.7	3.2	3.3	1.9
Solar Radiation (MJ/m^2^)	3.07	2.57	1.88	0.92

**Table 6:** Summary of field-test configurations used for simulated rainfall.

Setup	Description	Construction Area (m^2^)	Test Area (m^2^)	Simulated Rainfall (mm/h)
A	Conventional PP	124	80	15.63
B	LTPP with integrated storage	88		
C	LTPP-only	80		

**Table 7:** Drainage conditions used to evaluate theoretical storage of the LTPP system.

Drainage Rate (mm/h)	$D_{t}$ (mm/10 min)	Scenario Description	Condition
0	0	Static storage	No-drainage condition; test pure storage demand.
2	0.33	Low drainage	Conservative slow drawdown/low-permeability condition.
5	0.83	Moderate drainage	Partial-infiltration condition.
15	2.50	Design-threshold drainage	Minimum full-infiltration threshold commonly used for no-underdrain design [39].
30	5.00	High drainage	Highly permeable subgrade or efficient drainage condition

**Table 8:** Cumulative rainfall depth by duration and corresponding storage utilization relative to the 104 mm storage depth of the LTPP system.

Duration	Cumulative Rainfall Depth (mm)	Storage Utilization (%)
10 min	2	1.92
1 h	13	12.50
3 h	72	69.23
6 h	85	81.73
9 h	137	131.73
12 h	179	172.12
24 h	196	188.46

**Table 9:** Delayed-drainage scenario analysis of the LTPP system under the rainfall event using different effective drainage rates.

Drainage (mm/h)	Max. Stored Depth (mm)	Utilization (%)	Overflow Depth (mm)	Final Stored Depth (mm)	Emptying Time (h)
0	104	100	92	104	Not applicable
2	104	100	52	95.7	47.8
5	104	100	14.5	60.7	12.1
15	34.5	33.2	0	0	0
30	15	14.4	0	0	0

## Data Availability

The raw data supporting the conclusions of this article will be made available by the authors on request.

## Abbreviations

The following abbreviations are used in this manuscript:

LTPP	Low-tortuosity permeable pavement
PP	Conventional permeable pavement
UHI	Urban heat island
BA	Bottom ash
